# Urinary Ascites following Mini Lap Tubectomy: A Rare Occurence

**DOI:** 10.5402/2011/287102

**Published:** 2011-05-30

**Authors:** Pushpa Dahiya, Anshu Paul, Satish Dalal

**Affiliations:** ^1^Department of Obstetrics and Gynaecology, 2/7J Medical Campus, Pt. B.D. Sharma PGIMS, Rohtak 124001, Haryana, India; ^2^Department of Obstetrics and Gynaecology, 32/11J Medical Campus, Pt. B.D. Sharma PGIMS, Rohtak 124001, Haryana, India; ^3^Department of Surgery, Pt. B.D. Sharma PGIMS, Rohtak 124001, Haryana, India

## Abstract

Iatrogenic bladder injury is a known complication of laparoscopic and gynecological surgeries with an incidence of 1.5 per 1000 cases. Urinary ascites is a result of undiagnosed iatrogenic bladder injury during pelvic surgeries. We report a rare case of urinary ascites following mini lap tubectomy on the eighth postoperative day. After the diagnosis was made, conservative management was done for the patient, to which she successfully responded.

## 1. Introduction

A 28-years-old para three was referred to a tertiary care centre from a primary health centre on the eighth postoperative day of interval sterlization with complaints of gradually increasing abdominal distension causing respiratory difficulty, drowsiness, and inability to accept orally. Patient had undergone mini lap tubectomy 8 days back and was discharged six hours following surgery in good general condition. Patient reported back in 48 hours with distension of abdomen and persistent abdominal pain for which supportive treatment in the form of intravenous fluids, analgesics, and enema was given. As there was no relief of symptoms, patient was referred to tertiary care centre on the eighth postoperative day. On admission her general condition was poor. She was pale, dyspneic, and was unable to lie in dorsal position. On general physical examination, patient was febrile and had persistent tachycardia. Abdominal examination revealed generalized distension, tenderness, induration of wound, free fluid, and absent bowel sounds. After putting Foleys catheter only 100 cc of clear urine was drained. On pervaginal examination cervix and vagina was normal and uterus was felt floating with fullness in all fornices. Ultrasound was done which confirmed significant free fluid in abdomen. Abdominal paracentesis revealed pale yellow colored fluid which was coming freely.

Haemogram, biochemical, renal, and liver parameters were normal. Ascitic fluid on examination showed 10–15 polymorphs and creatinine levels of 590 *μ*mol/mL. Under local anesthesia, abdominal drain was inserted through which 1.75 litres of fluid was drained. Patient started showing signs of improvement with relief of distension, pain, and dyspnoea.

Suspecting it to be a case of iatrogenic bladder injury, surgery consultation was taken. IV urogram showed spillage of urine in the peritoneal cavity. Considering that the leak/bladder injury was small, decision for conservative management was taken and no bladder repair was done. Foleys catheterization was kept for 21 days. Abdominal drain was removed after 72 hours following nil output. Patient was kept under observation for 48 hours following catheter removal to watch for any signs of distension and pain. Patient was then discharged and kept on followup for three months.

## 2. Discussion

The incidence of bladder injury is 6% during obstetric procedures, 1.8% during caesarean section, and 1.5% during gynecological surgeries per 1000 cases [1, 2]. The anatomic proximity of the reproductive tract and lower urinary tract predisposes them to iatrogenic trauma during obstetric and gynecological surgeries. Bladder and distal ureters are most commonly involved organs [3]. Risk factors for bladder injuries include previous surgical interventions, pelvic inflammatory disease, endometriosis, anatomic anomalies, and pelvic adhesions [4]. Thus though iatrogenic ureteric and bladder injuries are globally rare they are liable to occour due to inherent anatomic and pathological factors in the pelvis. Iatrogenic bladder injuries have been reported in major laparoscopic and gynecological surgeries worldwide like total abdominal hysterectomy, vaginal hysterectomy, caesarean section, cancer surgeries, ovariotomy, but to our knowledge, no case has been reported till date where iatrogenic bladder injuries occurred during mini lap tubectomy resulting in urinary ascites. Injury might have occurred due to small abdominal incision of mini lap, less visibility, and less expertise of the operating surgeon. Simple procedures such as emptying the bladder preoperatively or inserting a foley's catheter, monitoring the urine colour and output, having a good surgical exposure and following proper techniques can prevent iatrogenic bladder injuries intraoperatively. Also when in doubt regarding iatrogenic bladder injury, methylene blue test should be done and if indicated surgical opinion should be taken regarding cystoscopy and suprapubic telescopy. Repair of the bladder injury at the time of primary surgery is easier, more successful, and less morbid for the patient and medicolegally advantageous for the surgeon. Delayed diagnosis is suspected when postoperatively there is oliguria, haematuria, elevated urea/creatinine ratio, lower abdominal pain, distension, paralytic ileus, or urinary ascites as was seen in the present case. Stress cystography, ultrasonography, abdominal CT scan, CT cystography, cystoscopy, or retrograde pyelography will be of help in diagnosing and localizing bladder injury [3].

Thus unexplained ascites and decreased renal function in a previously healthy person with a recent history of pelvic surgery should raise the suspicion of intraperitoneal bladder leak. A very high creatinine level in ascitic fluid is diagnostic of urinary ascites. An ascitic fluid creatinine: serum creatinine ratio >1 is highly suggestive of intraperitoneal urine leak [5]. In most of the cases of urinary ascites reported, following gynecological surgeries, repair of bladder injuries and in some cases ureteric injuries, surgical repair was done, once the diagnosis was made [4]. But in the present case, since the leak was small, decision for conservative management was successful.

## 3. Conclusion

Thus it is necessary to have a high index of suspicion to diagnose ureteric or bladder injuries in patients presenting with unexplained ascites, deterioration of renal function in a previously healthy person with a recent history of pelvic surgery. Imaging is essential for diagnosis of iatrogenic bladder injuries. A high creatinine level in the ascitic fluid and an ascitic fluid creatinine: serum creatinine ratio >1 is suggestive of intraperitoneal leak [4]. Thus in conclusion to avoid iatrogenic bladder injuries, the gynecologist should identify high-risk cases, evaluate them preoperatively, and prepare accordingly. Whenever in doubt of bladder injury intraoperatively, methylene blue test or surgical opinion should be taken and, if indicated, cystoscopy should be done.
